# Mechanism by which SAHA regulates HLA-E expression via the endoplasmic reticulum stress-related PERK/ATF4/CHOP pathway in neuroblastoma

**DOI:** 10.3389/fimmu.2026.1741513

**Published:** 2026-03-25

**Authors:** Zhuoran Li, Xi Zhen, Chenggong Zeng, Yan Mao, Zhiqing Wei, Zijun Zhen

**Affiliations:** 1 State Key Laboratory of Oncology in South China, Guangdong Provincial Clinical Research Center for Cancer, Sun Yat-sen University Cancer Center, Guangzhou, China; 2 The Pennsylvania State University State College, PA, United States

**Keywords:** endoplasmic reticulum stress (ERS), human leukocyte antigen E (HLA-E), immune evasion, neuroblastoma, suberoylanilide hydroxamic acid (SAHA)

## Abstract

**Background:**

Human leukocyte antigen E (HLA-E) plays a role in tumor immune escape and is associated with poor prognosis in neuroblastoma (NB). This study aimed to investigate the regulatory effect of suberoylanilide hydroxamic acid (SAHA) on HLA-E expression via the PERK/ATF4/CHOP pathway in NB.

**Methods:**

A high HLA-E expression model in NB cells was established by stimulation with interferon-gamma (IFN-γ). The effects of SAHA on NB cell proliferation and migration were evaluated. In addition, the influence of SAHA on the PERK/ATF4/CHOP signaling pathway and HLA-E expression at the mRNA and protein levels was analyzed. Bioinformatics analysis was performed using data from the TARGET and Gene Expression Omnibus (GEO; GSE85047) databases to identify prognostic genes associated with NB.

**Results:**

Stimulation with IFN-γ successfully induced high HLA-E expression in NB cells. SAHA significantly suppressed NB cell proliferation and migration and downregulated HLA-E expression at both the mRNA and protein levels. Analysis of the TARGET database revealed that the prognosis of patients with NB was closely related to the expression levels of endoplasmic reticulum stress (ERS)-related proteins, particularly PERK and HLA-E. This association was validated using the GEO dataset GSE85047. Moreover, SAHA inhibited the expression of ERS pathway proteins, including PERK and CHOP, in NB cell lines.

**Conclusion:**

This study demonstrated that SAHA downregulates HLA-E expression by inhibiting the PERK/ATF4/CHOP pathway, offering new insights into the regulation of tumor proliferation, migration, and immune evasion in NB.

## 1 Introduction

Neuroblastoma (NB) is the most common extracranial solid tumor in children, accounting for approximately 8% of all pediatric malignancies and 10–15% of pediatric cancer-related deaths. More than 60% of patients present with high-risk disease at diagnosis (1). Although treatment outcomes for high-risk NB have improved through multimodal strategies, including immunotherapy, relapse still occurs in approximately one-third of patients. The treatment of relapsed or refractory NB remains a significant clinical challenge (2, 3). The molecular mechanisms underlying this aggressive phenotype remain poorly understood.

Emerging evidence suggests that human leukocyte antigen E (HLA-E) plays a role in tumor progression. HLA-E is overexpressed in various adult cancers (4–12), as well as in NB (13–17), and is associated with poor prognosis (4–11, 18). Tumor-expressed HLA-E binds to inhibitory receptors such as CD94/NKG2A on natural killer (NK) cells and cytotoxic T lymphocytes, suppressing their cytolytic function (12) and facilitating immune escape. Given that CD94 is widely expressed on NK cells, tumor-derived HLA-E may contribute to widespread immune suppression by NK cells (19).

However, the mechanisms that regulate HLA-E expression are not yet fully understood. Previous studies have demonstrated that interferon-gamma (IFN-γ) upregulates HLA-E expression via signaling pathways such as STAT1 and GATA-1 (20, 21), while heat shock proteins have been shown to downregulate HLA-E (22). Endoplasmic reticulum stress (ERS) has been implicated in the pathogenesis of multiple diseases (23). Sustained ERS activates the unfolded protein response (UPR), potentially leading to apoptosis. The PERK/ATF4/CHOP axis is one of the three major UPR signaling branches and has been shown to play a role in various tumor types, including NB. However, the role of ERS in the regulation of HLA-E expression remains unclear.

Suberoylanilide hydroxamic acid (SAHA), a broad-spectrum histone deacetylase (HDAC) inhibitor, has demonstrated antitumor activity in a wide range of cancers (43–50). HDACs have been shown to regulate ATF4 (24), and degradation of HLA-E protein at the cell surface is closely associated with ERS (13). Based on these findings, we hypothesize that SAHA modulates HLA-E expression in NB cells via ERS-related signaling pathways, thereby potentially reversing immune escape in NB. This study aims to provide a theoretical basis for developing novel therapeutic strategies targeting high-risk NB.

## 2 Materials and methods

### 2.1 Cell culture and drug incubation

Four human NB cell lines were used in this study: SK-N-BE (2) (MYCN-amplified), SK-N-SH (non-MYCN-amplified), KELLY(MYCN-amplified) and CHP212 (MYCN-amplified). SK-N-BE (2), KELLY and SK-N-SH cells were obtained from PROCELL (Wuhan, Hubei, China), and CHP212 cells were purchased from COBIOER (Nanjing, Jiangsu, China).

SK-N-BE (2) and CHP212 cells were cultured in MEM-F12 medium supplemented with 10% fetal bovine serum, 1% non-essential amino acids, 1% sodium butyrate, 10,000 U/mL penicillin, and 10,000 μg/mL streptomycin. SK-N-SH cells were cultured in MEM medium under the same supplementation. All media and reagents were sourced from Gibco (Waltham, MA, USA).

Cells were routinely subcultured using 0.25% trypsin-EDTA solution when they reached approximately 80% confluence. The drugs used for treatment were IFN-γ (MedChemExpress, New Jersey, USA) and SAHA (Vorinostat) (Selleck, Houston, USA).

### 2.2 Cell migration assay

Cells were trypsinized and resuspended in serum-free medium, then seeded into six-well plates at a density of 1 × 10^6^ cells/mL. After overnight incubation at 37 °C in a humidified atmosphere with 5% CO_2_, a scratch was made in each well using a sterile pipette tip. Fresh serum-free medium was added, and images were captured at 0, 24, and 72 h using a microscope to monitor wound closure. The wound distance was measured, and group differences were analyzed statistically.

### 2.3 Cell proliferation assay

Cell proliferation was assessed using the Cell Counting Kit-8 (CCK-8) assay. Cells were seeded in 96-well plates at a density of 10,000 cells per well and incubated overnight at 37 °C in 5% CO_2_. After 24 h, cells were treated with varying concentrations of SAHA or control drugs.

After 48 h, the medium was removed, and 100 µL of serum-free medium containing 10% CCK-8 reagent (APExBIO, Houston, TX, USA) was added to each well. The plates were incubated for 4 h at 37 °C, and absorbance was measured at 450 nm using a microplate reader (M200, Tecan). Data were analyzed using GraphPad Prism 8.0. Cell proliferation was calculated based on absorbance values, and a dose-response curve was plotted. The concentration that inhibited 50% of cell proliferation (IC_50_) was calculated accordingly.

### 2.4 Quantitative real-time PCR

The RNA Rapid Extraction Kit was purchased from ES Science (Shanghai, Jiangsu, China). Primers were synthesized by Shanghai Generay. The primer sequences are listed in **Table 1**. A 10 µL PCR reaction mixture was prepared by sequentially adding reagents according to the instructions provided with the reverse transcription kit. The Fast All-in-One RT Kit (with cDNA Remover) was obtained from ES Science (Shanghai, Jiangsu, China). The PCR reaction solution (**Table 2**) was prepared in the dark. The mixture was then transferred to ice in preparation for subsequent qRT-PCR, which was conducted using Power SYBR Green qPCR Mix (Dongsheng Biological Technology Co., Ltd., Guangzhou, Guangdong, China).

**Table 1:** Primers used for qRT-PCR.

Gene	Forward primer (5’-3’)	Reverse primer (5’-3’)
*β-actin*	ATTGGCAATGAGCGGTTC	CGTGGATGCCACAGGACT
*HLA-E*	TTCCGAGTGAATCTGCGGAC	GTCGTAGGCGAACTGTTCATAC
*PERK*	AGACATGGAAACGAGAGCCG	ACTTTCCAGTCAGCAACCGA
*ATF4*	GCAAAACAAGACAGCAGCCA	ACTTTCCAGTCAGCAACCGA
*CHOP*	TTCACCACTCTTGACCCTGC	CTCCTTCATGCGCTGCTTTC

**Table 2:** Reagent ratios for reverse transcription.

Component	Size (μL)
SYBR Green qPCR Mix	5.0
Upstream primer (μM)	0.2
Downstream primer (μM)	0.2
PCR model cDNA	1.0
DEPC water	3.6
Total	10.0

### 2.5 Western blot

Cells were digested and transferred into labeled centrifuge tubes, then washed with precooled PBS. They were lysed and stored at –80 °C for future use. The Protein Quantification Kit was purchased from Thermo Fisher (Waltham, MA, USA). The working solution was prepared by mixing Solution A and Solution B at a ratio of 50:1. The standard protein used was bovine serum albumin, with a stock concentration of 2,000 μg/mL. Absorbance was measured at 562 nm using a multifunctional microplate reader (M200, Tecan).

Loading buffer was added to 20 µg of protein sample, and the mixture was heated to denature the proteins. Proteins were separated on a 12% separating gel and a 5% stacking gel using an electrophoresis apparatus. Electrophoresis was continued until the protein marker neared the bottom of the separating gel. Proteins were transferred onto a PVDF membrane, which was then washed and blocked with 5% skim milk at room temperature for 1 h to prevent non-specific binding. After incubation with primary antibodies, the membrane was washed and incubated with a secondary antibody at room temperature for 1 h. The membrane was treated with SuperSignal West Atto and imaged using a chemiluminescence imaging system (Bio-Rad). Tubulin was used as a loading control for normalization.

### 2.6 Acquisition of gene expression and statistical analysis

Gene expression profiles and clinical information for NB tissue samples were obtained from the TARGET database (https://www.cancer.gov/ccg/research/genome-sequencing/target/studied-cancers/neuroblastoma) and the Gene Expression Omnibus (GEO) database (https://www.ncbi.nlm.nih.gov/geo/query/acc.cgi?acc=GSE85047). The TARGET database included 151 patients with NB (version: 07-23-2019), and the GSE85047 dataset contained 283 patients (latest update: February 18, 2019). After downloading the data on December 12, 2023, and removing patients with incomplete clinical information, 114 and 272 patients from the TARGET and GSE85047 datasets, respectively, were included in the analysis.

To identify ERS-related and prognosis-related signatures for survival prediction, univariate Cox regression and Log-rank analyses were used to screen for prognosis-related genes in the TARGET database (cut-off p < 0.05). ERS-related genes were selected using the KEGG, GO, and GeneCards databases. Six overlapping genes were used to construct a risk signature via the least absolute shrinkage and selection operator (LASSO) regression model. Three key genes were identified, and their prognostic performance was evaluated using the ROC curve, Kaplan–Meier survival analysis, and multivariate Cox regression. Gene expression correlations were assessed using Pearson correlation coefficients. All statistical analyses were conducted using R software. A p-value < 0.05 was considered statistically significant.

## 3 Results

### 3.1 IFN-γ induces HLA-E expression in NB

**Figure 1:**
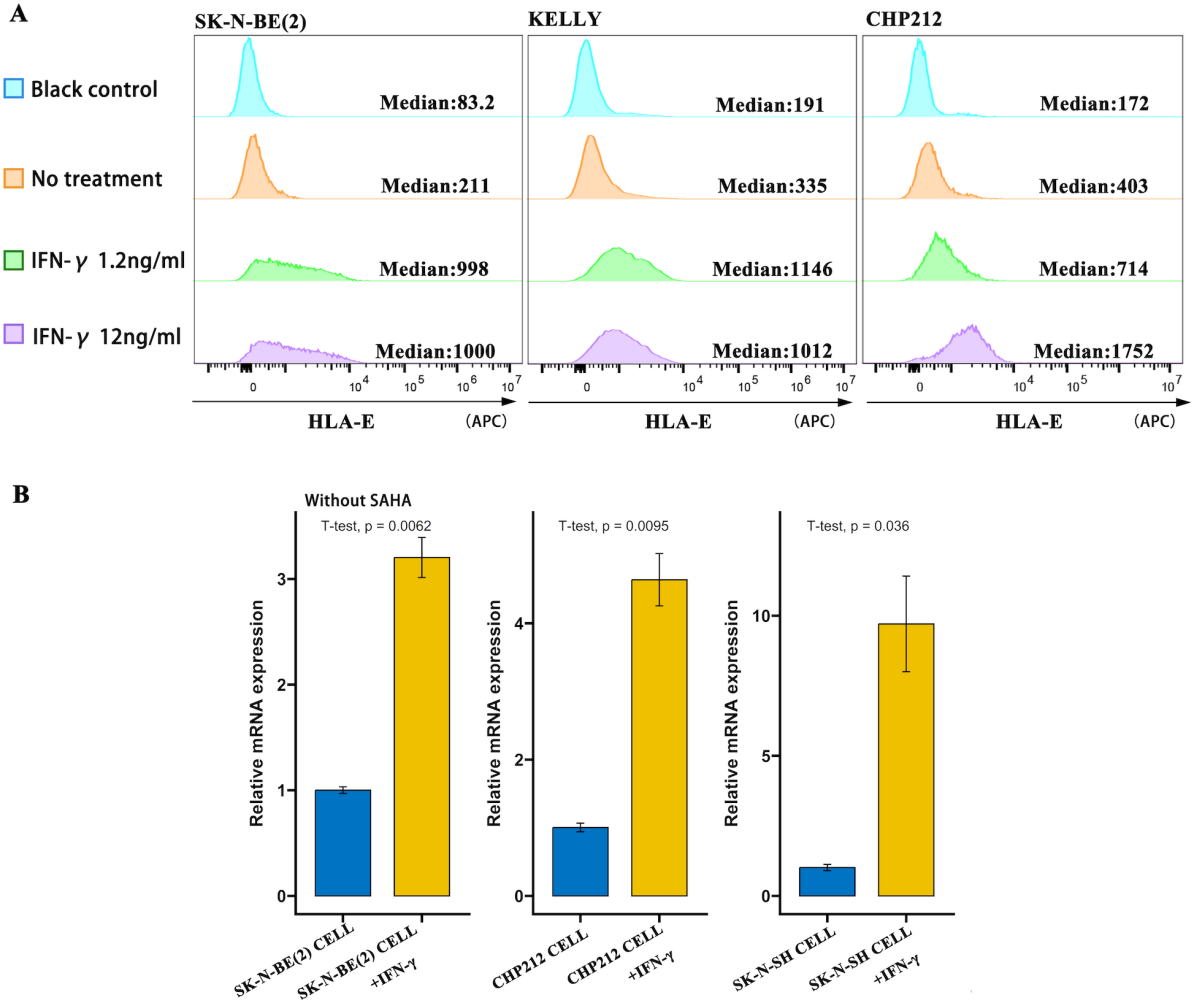
IFN-γ induces HLA-E expression in NB cell lines. **(A)** HLA-E expression levels were analyzed by flow cytometry in SK-N-BE(2), KELLY, and CHP212 cells after incubation with various concentrations of IFN-γ. **(B)** HLA-E mRNA levels increased in multiple NB cell lines following IFN-γ incubation, as measured by qRT-PCR.

To investigate whether and how IFN-γ affects HLA-E expression in NB, HLA-E levels were measured at both the RNA and protein levels using qRT-PCR and flow cytometry, respectively. Without IFN-γ stimulation, the proportion of HLA-E(+) cells in *MYCN*-amplified NB cell lines SK-N-BE(2), KELLY, and CHP212 was 3.72%, 11.4%, and 11.5%, respectively. Following incubation with increasing concentrations of IFN-γ, the proportion of HLA-E(+) cells increased significantly. At 12 ng/mL IFN-γ, the proportions rose to 46.6% (MFI = 1,000), 50.8% (MFI = 1,012), and 81.0% (MFI = 1,752) in SK-N-BE(2), KELLY, and CHP212 cells, respectively—substantially higher than the controls (**Figure 1A**). After IFN-γ incubation, the mRNA levels of HLA-E also increased significantly in both *MYCN*-amplified NB cells (SK-N-BE(2) and CHP212) and *MYCN* non-amplified NB cells (SK-N-SH) (p < 0.05) (**Figure 1B**).

### 3.2 SAHA inhibits cell proliferation and migration in NB

**Figure 2:**
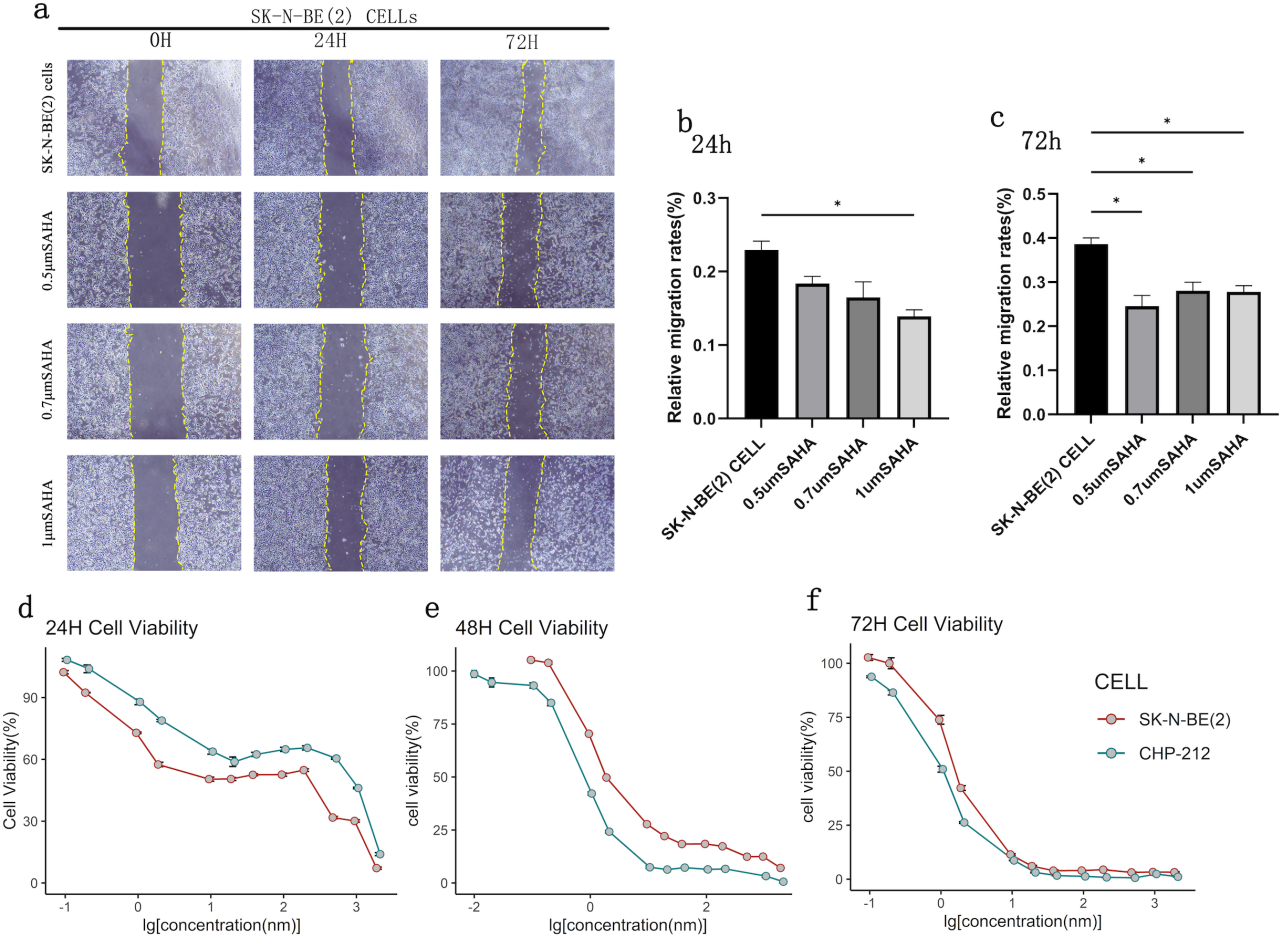
SAHA reduces the migration and proliferation of NB cell lines. **(A)** Cell scratch test of SK-N-BE(2) cells; **(B)** Relative migration rates of SK-N-BE(2) cells after incubation with SAHA for 24 h; **(C)** Relative migration rates of SK-N-BE(2) cells after incubation with SAHA for 72 h; **(D–F)** Cell viability of SK-N-BE(2) and CHP212 cells after incubation with SAHA for 24, 48, and 72 h, respectively. *: *P* < 0.05.

After 24 h of SAHA incubation, the relative migration rate of the control group was 22.93%, whereas in the 0.5, 0.7, and 1 μM treatment groups, the rates were 18.37%, 16.50%, and 13.91%, respectively, in SK-N-BE(2) cells. After 72 h of SAHA incubation, the relative migration rate of the control group was 38.62%, while those of the treatment groups were 24.57% (0.5 μM), 28.05% (0.7 μM), and 27.80% (1 μM) in SK-N-BE(2) cells. The relative migration rates significantly decreased following SAHA treatment at both 24 and 72 h (p < 0.05) (**Figures 2A–C**).

To elucidate the effects of SAHA on NB cells, we assessed the viability of SK-N-BE(2) and CHP212 cell lines exposed to varying concentrations of SAHA at 24, 48, and 72 h (**Figures 2D–F**). Our results demonstrated that NB cell viability decreased in a concentration-dependent manner with increasing SAHA exposure. In the 48 h treatment groups, the IC_50_ values for SK-N-BE(2) and CHP212 cells were 1.21 μM and 3.85 μM, respectively.

### 3.3 SAHA reduces the expression of HLA-E in NB

**Figure 3:**
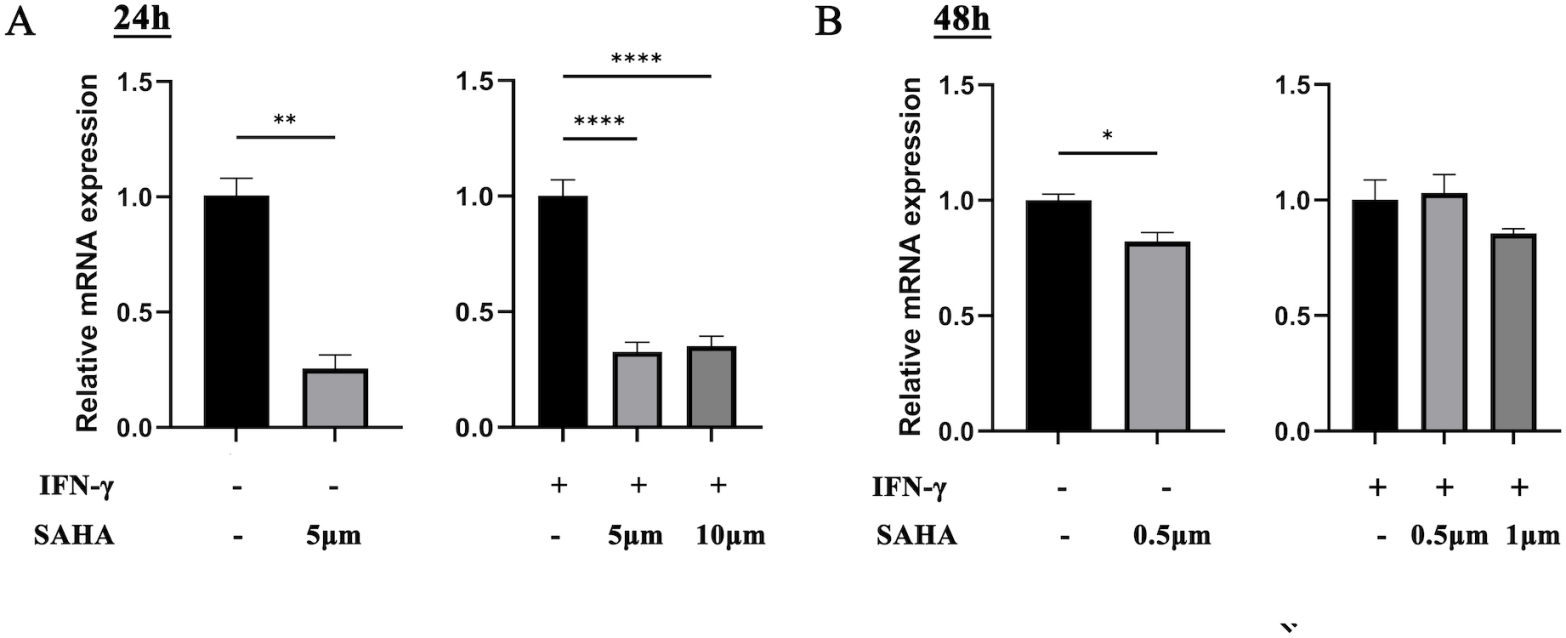
The mRNA level of SK-N-BE(2) cells after exposure to SAHA. **(A)** SAHA only or combined with IFN-γ cultured for 24 h in SK-N-BE(2) cells. **(B)** SAHA only or combined with IFN-γ cultured for 48 h in SK-N-BE(2) cells. *: *P* < 0.05, **: *P* < 0.01, ***: *P* < 0.001, ****: *P* < 0.0001.

To investigate the relationship between SAHA and HLA-E expression in NB, mRNA and protein levels of HLA-E were analyzed following SAHA treatment. The IC_50_ values of SAHA in SK-N-BE(2) cells at 24 and 48 h were 12.9 μM (95% CI: 8.89–18.67, R² = 0.90) and 1.20 μM (95% CI: 1.05–1.38, R² = 0.98), respectively. HLA-E mRNA expression was significantly suppressed by SAHA in SK-N-BE(2) cells after both 24 and 48 h, regardless of IFN-γ treatment (p < 0.05) (**Figures 3A, B**).

**Figure 4:**
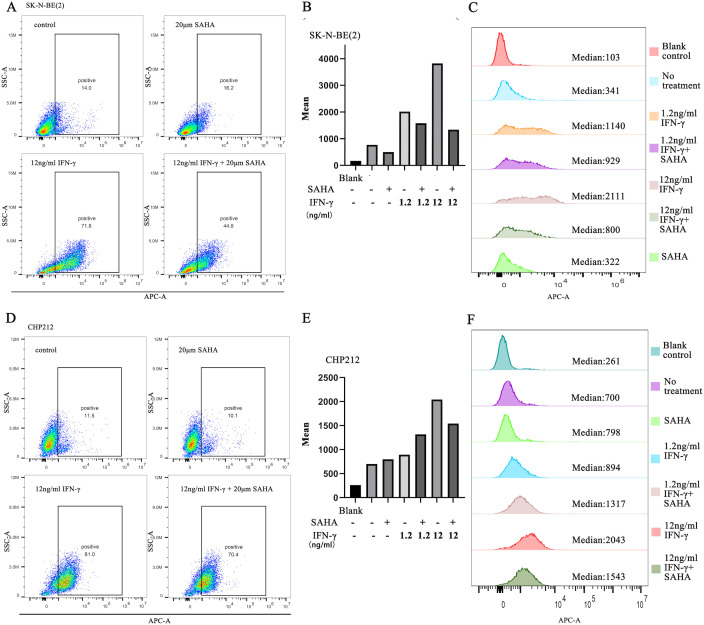
The mRNA level of IFN-γ-stimulated NB cells after exposure to SAHA. **(A–C)** Scatter plot, bar chart, and histogram of HLA-E in IFN-γ-stimulated SK-N-BE(2) cells after exposure to SAHA. **(D–F)** Scatter plot, bar chart, and histogram of HLA-E in IFN-γ-stimulated CHP212 cells after exposure to SAHA.

Without IFN-γ stimulation, the proportion of HLA-E(+) cells before and after SAHA treatment was similar: 14.0% (MFI = 341) and 16.2% (MFI = 322) in SK-N-BE(2) cells, and 11.5% (MFI = 700) and 10.1% (MFI = 798) in CHP212 cells, respectively. Following incubation with 12 ng/mL IFN-γ, the HLA-E(+) population increased to 71.8% (MFI = 2111) and 81.0% (MFI = 2043) in SK-N-BE(2) and CHP212 cells, respectively. However, when co-treated with SAHA and IFN-γ, the HLA-E(+) population decreased to 44.8% (MFI = 800) and 70.4% (MFI = 1543), respectively (**Figure 4**).

### 3.4 The correlation between ERS-related proteins, HLA-E, and the prognosis of NB

**Figure 5:**
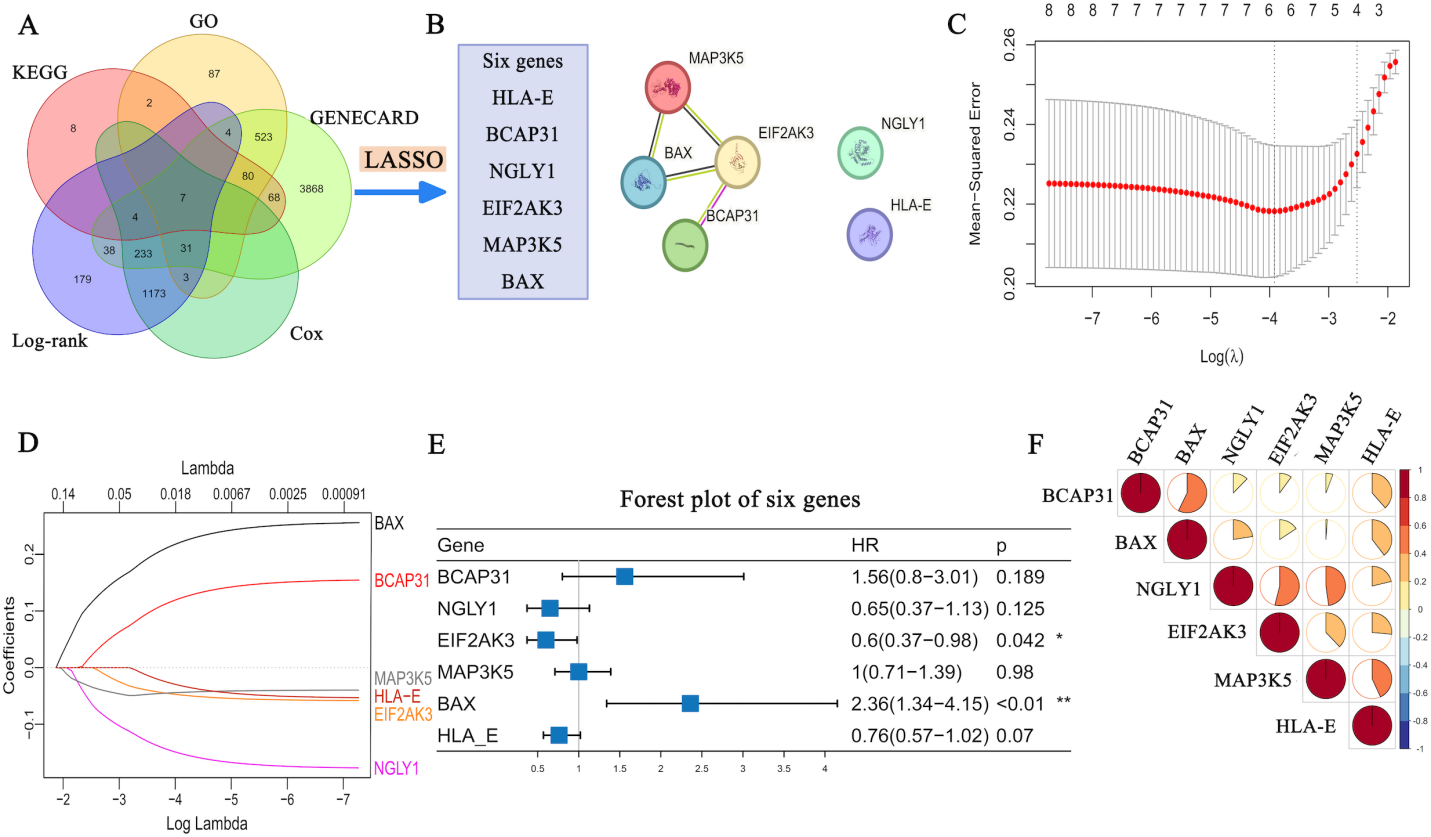
The association between the expression of PERK/EIF2AK3, HLA-E, and BAX genes and the prognosis of patients with NB. **(A)** Prognosis-related genes acquired by Cox univariate and log-rank analysis. After intersection, 1451 genes were determined as prognosis-related (p < 0.05). There were 169, 737, and 4,856 genes selected from the KEGG, GO, and GeneCards databases, respectively. After intersection, 87 ERS-related genes were selected. Prognosis-related and ERS-related genes were intersected to examine their relationship with HLA-E and identify six hub genes via LASSO analysis. **(B)** Relationships among six important genes; **(C)** Change in mean squared error of LASSO regression with log(λ); **(D)** Change in regression coefficients with log(λ); **(E)** Multivariate Cox regression (concordance index: 0.72, global p-value [log-rank]: 1.101e-05); **(F)** Correlation heatmap of the six important genes. *: Indicates *P* < 0.05, **: Indicates *P* < 0.01.

ERS-related genes (n = 87) were selected from the GO, KEGG, and GeneCards databases, and prognosis-related genes (n = 1,451) were identified from the TARGET- NB cohort (n = 114) by univariate Cox regression analysis and log-rank test (p < 0.05). After intersection, seven genes were retained, including HLA-E, the primary gene of interest (**Figure 5A**).

Using LASSO regression with the minimum criteria, six genes were selected: *HLA-E, BCAP31, NGLY1, EIF2AK3, MAP3K5*, and *BAX.* The relationships among these genes are shown in **Figure 5B** and **5F**. Ultimately, three robust prognostic genes were retained following multivariate Cox regression: *PERK*, *HLA-E*, and *BAX*. These genes are highly associated with NB progression (**Figures 5C–F**).

The risk scores were calculated based on the expression of three genes using the following formula:

Risk score = (−0.7209 × EIF2AK3 expression) + (1.0348 × BAX expression) + (−0.2522 × HLA-E expression).

**Figure 6:**
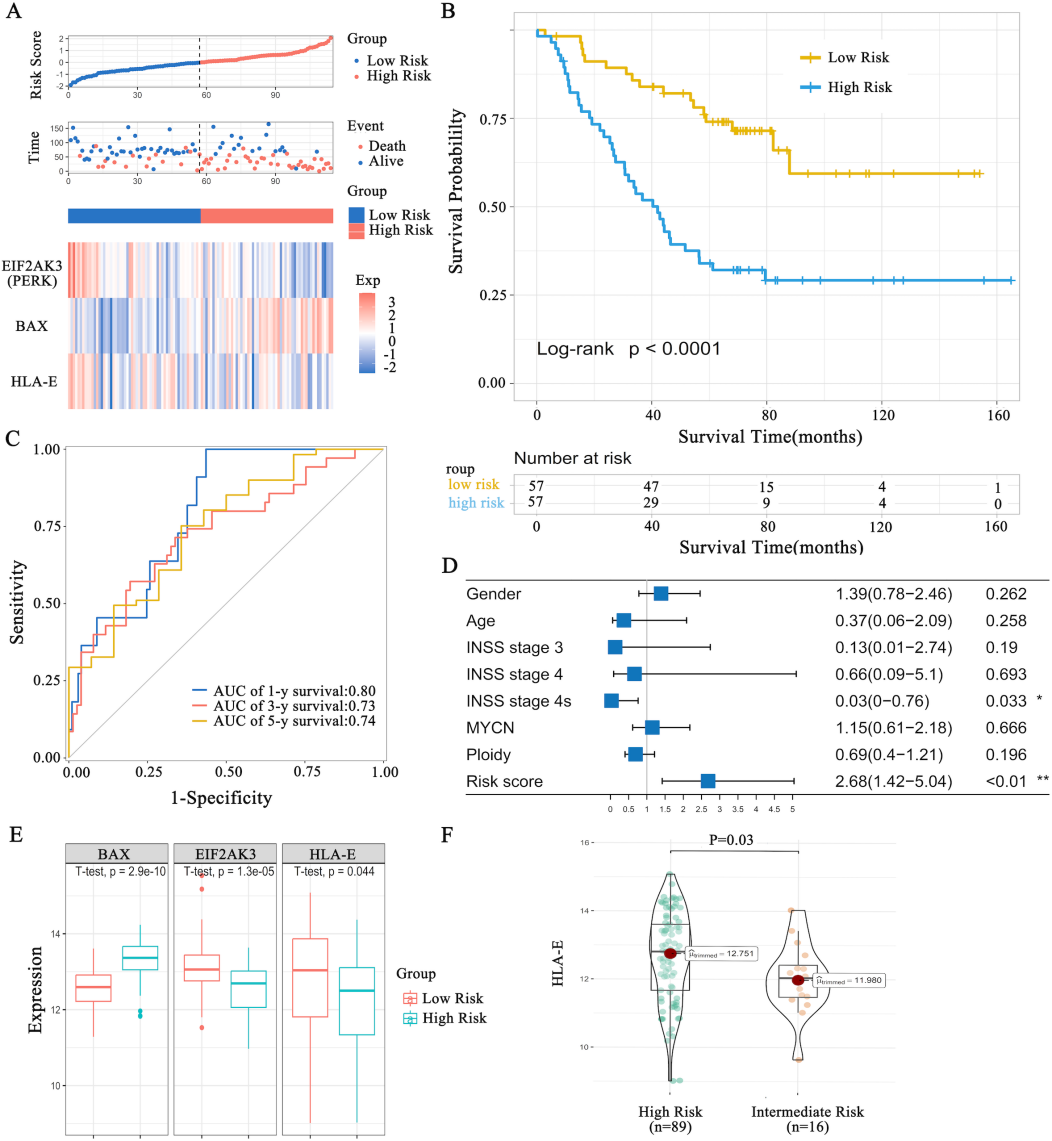
The risk score and prognosis of patients with NB. **(A)** Patients with NB were divided into the low-risk and high-risk groups according to the median risk score. **(B)** Survival analysis of patients with NB stratified into low- and high-risk groups. **(C)** Time-dependent ROC curve of patients with NB. **(D)** Cox multivariate analysis of NB risk factors. **(E)** Expression levels of PERK (EIF2AK3), HLA-E, and BAX in different groups. **(F)** HLA-E expression is higher in the COG high-risk group than in the intermediate-risk group. *: Indicates *P* < 0.05, **: Indicates *P* < 0.01.

Patients were divided into high-risk (n = 57) and low-risk (n = 57) groups according to the median risk score. The high-risk group exhibited a considerably worse prognosis (**Figures 6A**, **B**). The gene expression heatmap displayed distinct expression levels of the three selected hub genes in the high-risk and low-risk groups (**Figure 6A**). The 5-year overall survival (OS) rates for the high-risk and low-risk groups were 34.0% ± 6.3% and 74.0% ± 6.0%, respectively (**Figure 6B**). Time-dependent ROC curves were generated to evaluate the predictive value of the three genes for OS, showing 1-, 3-, and 5-year AUCs of 0.80 ± 0.05, 0.73 ± 0.05, and 0.74 ± 0.07, respectively (**Figure 6C**). It is important to note that the negative coefficient for HLA-E in this multi-variable model indicates that, after accounting for the effects of other clinical risk factors, higher HLA-E expression is independently associated with a more favorable prognosis. This contrasts with its univariate association with high-risk groups and highlights the context-dependent prognostic role of HLA-E in neuroblastoma (**Figure 6E, F**).

According to Cox multivariate analysis, the risk score was an independent prognostic factor for NB (HR = 2.68, 95% CI: 1.42–5.04, *p* < 0.01) (**Figure 6D**). In the TARGET NB cohort, patients were stratified based on the risk classification system of the Children’s Oncology Group (COG) (14). Patients in the high-risk group exhibited significantly higher HLA-E expression than those in the intermediate-risk group (p = 0.03) (**Figure 6F**).

### 3.5 Validation of the three hub genes in the GEO cohort

**Figure 7:**
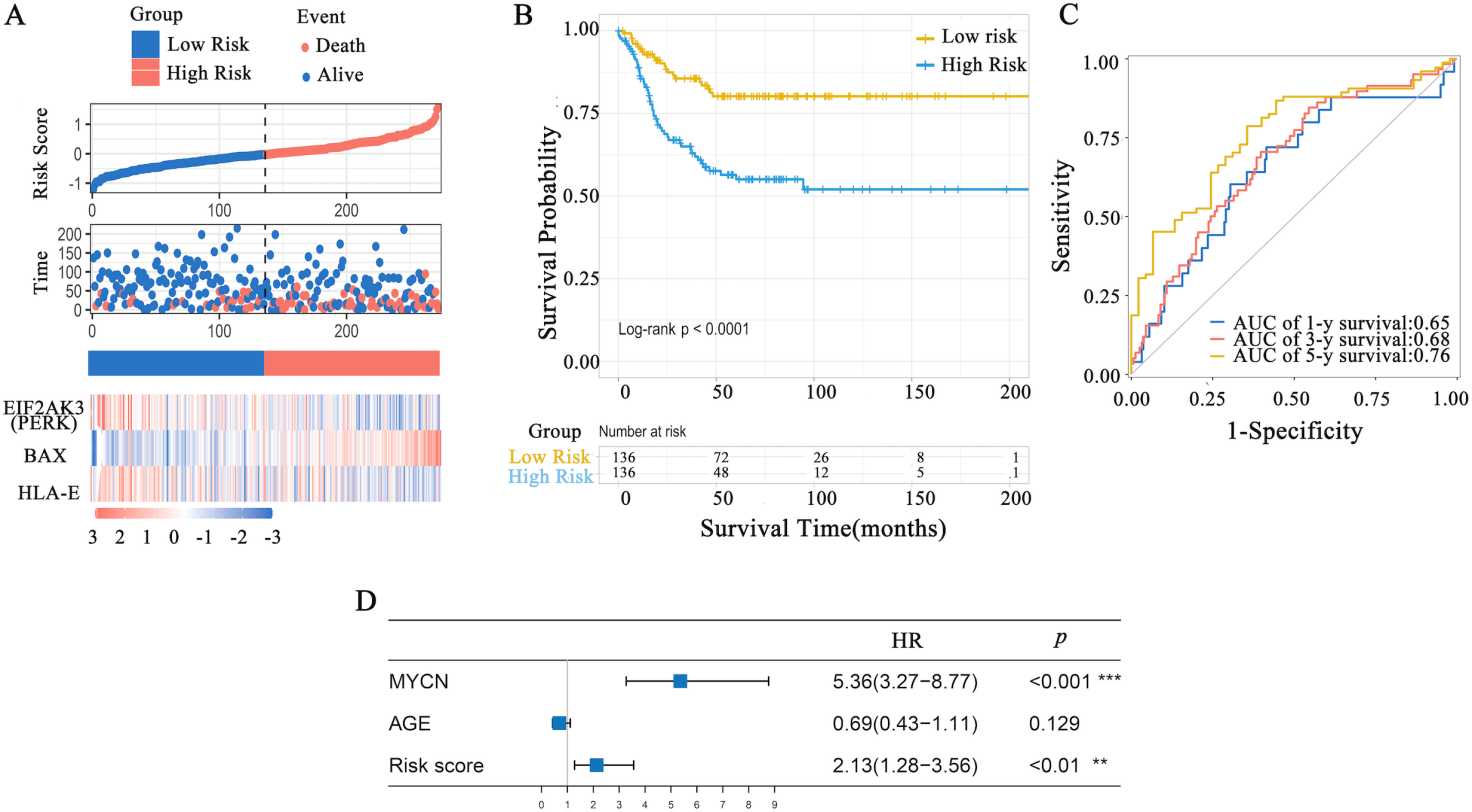
Validation of risk score efficacy using the GSE85047 dataset. **(A)** Risk model distribution and gene expression heatmap for the three key genes. **(B)** Survival analysis of patients stratified by risk score. **(C)** Time-dependent ROC curves for OS; AUCs for 1-, 3-, and 5-year survival were 65.18 ± 6.05%, 67.90 ± 4.08%, and 75.83 ± 4.51%, respectively. **(D)** Cox multivariate analysis of NB risk factors. **: Indicates *P* < 0.01, ***: Indicates *P* < 0.001.

The GEO dataset (accession number GSE85047, n = 272) was used as an external validation cohort. Risk scores for each sample were calculated using the same formula as for the TARGET cohort. The distribution of the OS-related risk model revealed distinct risk scores between the high- and low-risk groups, and the heatmap illustrated gene expression patterns, showing clear trends between the two groups (**Figure 7A**). The high-risk group showed a significantly poorer survival rate compared to the low-risk group (p < 0.01) (**Figure 7B**). The 5-year OS rates were 80.2 ± 3.8% (95% CI: 0.73–0.88) for the low-risk group and 56.4 ± 4.8% (95% CI: 0.47–0.67) for the high-risk group. Time-dependent ROC curves showed robust predictive value, with 1-, 3-, and 5-year AUCs of 0.65, 0.68, and 0.76, respectively (**Figure 7C**). Cox multivariate analysis further supported the independent prognostic significance of the risk model (**Figure 7D**).

### 3.6 SAHA regulates the expression of the PREK/ATF4/CHOP pathway in NB

**Figure 8:**
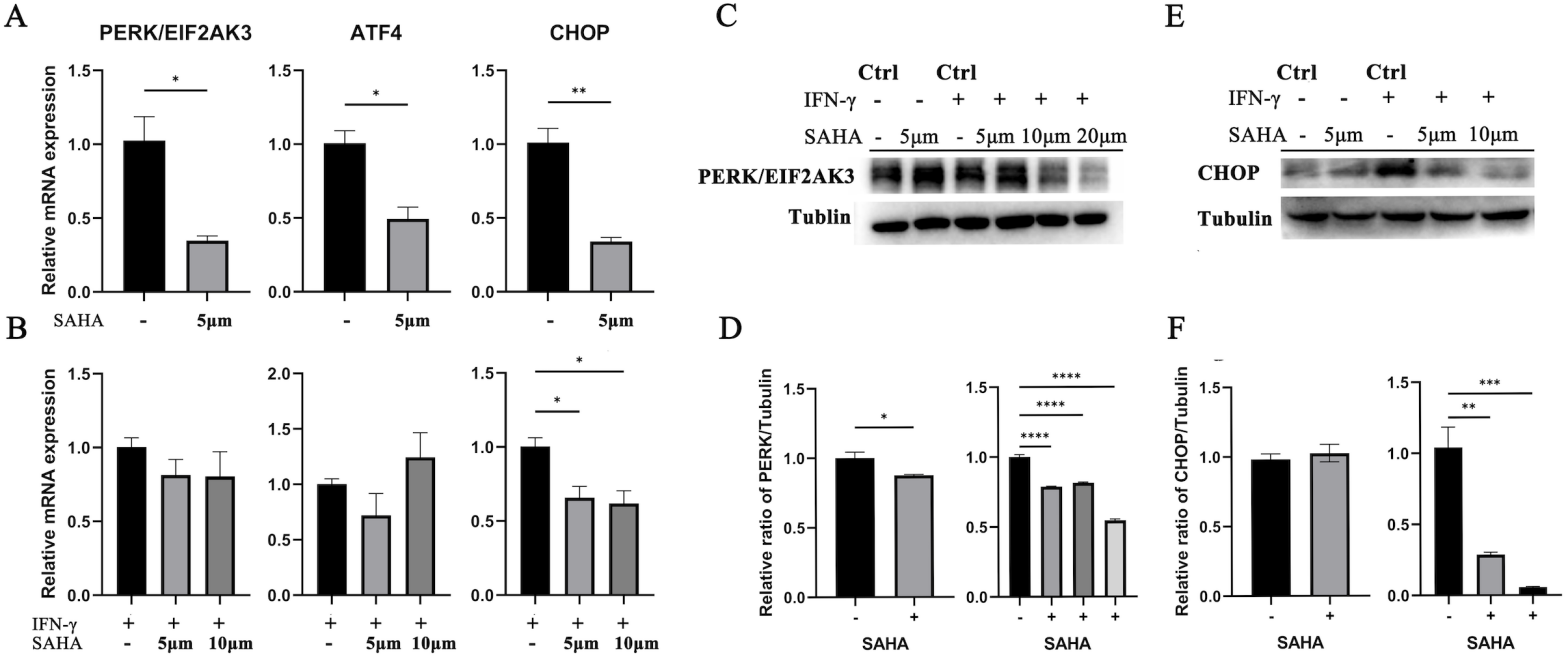
SAHA downregulated the mRNA levels of the PERK/ATF4/CHOP pathway. **(A)** In SK-N-BE(2) cells incubated with SAHA for 24 h, the mRNA levels of PERK/EIF2AK3, ATF4, and CHOP were significantly downregulated. **(B)** In cells stimulated with IFN-γ, SAHA significantly decreased the mRNA expression level of CHOP, while the mRNA levels of PERK/EIF2AK3 and ATF4 showed a trend toward downregulation near statistical significance. *p < 0.05, **p < 0.01. **(C–F)** Changes in protein levels after SAHA incubation. *: *P* < 0.05, **: *P* < 0.01, ***: *P* < 0.001, ****: *P* < 0.0001.

In SK-N-BE(2) cells treated with SAHA for 24 h, the mRNA levels of PERK/ATF4/CHOP pathway genes significantly decreased compared to the baseline (p < 0.05). In cells with HLA-E upregulated by IFN-γ, SAHA significantly downregulated CHOP mRNA (p < 0.05), while PERK expression approached statistical significance (**Figure 8B**).

Western blot analysis confirmed that 24 h of SAHA treatment led to a significant reduction in PERK and CHOP protein levels in SK-N-BE(2) cells (*p* < 0.05) (**Figures 8C–F**). PERK protein levels were notably reduced by SAHA alone or in combination with IFN-γ (*p* < 0.05). CHOP protein expression did not significantly change with SAHA alone but was significantly reduced with IFN-γ stimulation. These findings indicate that SAHA downregulates PERK and CHOP expression at both mRNA and protein levels.

**Figure 9:**
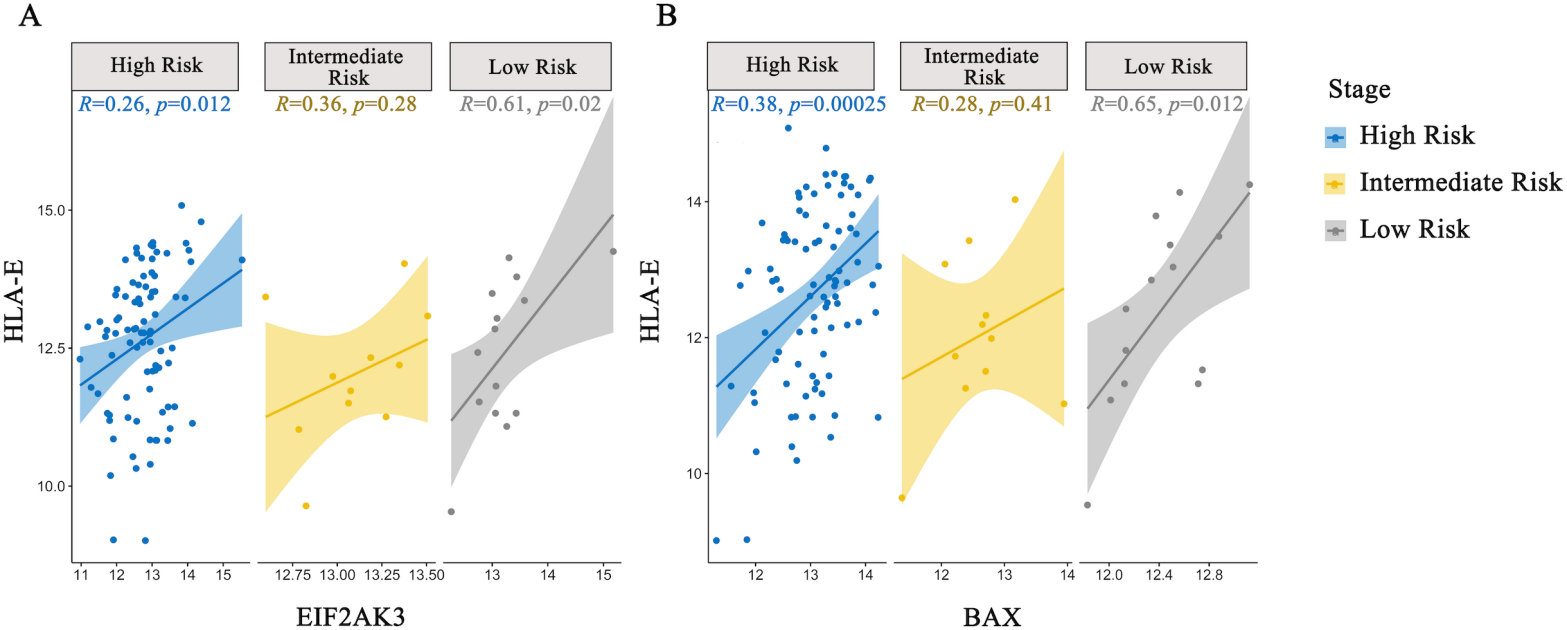
Pearson correlation analysis between PERK (EIF2AK3), BAX, and HLA-E. **(A)** HLA-E expression was positively correlated with PERK (EIF2AK3). **(B)** HLA-E expression was positively correlated with BAX.

To investigate the association between the PERK pathway and HLA-E expression in NB, we analyzed patient data from the TARGET database. HLA-E and PERK (EIF2AK3) expression were positively correlated in the COG low-, intermediate-, and high-risk groups, with Pearson correlation coefficients (R) of 0.26 (p = 0.01), 0.36 (p = 0.28), and 0.61 (p = 0.02), respectively. Similarly, HLA-E expression was positively correlated with BAX expression, with correlation coefficients of 0.38 (p < 0.01), 0.28 (p = 0.41), and 0.65 (p = 0.01), respectively (**Figure 9**).

## 4 Discussion

HLA-E plays a critical role in the development and progression of NB and adversely affects patient survival. This study aimed to elucidate the regulatory mechanism by which SAHA influences HLA-E expression in NB. We demonstrated that SAHA reduced cell proliferation and migration in NB cell lines regardless of MYCN amplification status, suggesting its therapeutic potential. Our findings are consistent with previous studies reporting the antitumor effects of SAHA in NB cell models and animal studies (16, 17), as well as in other tumor types such as melanoma, Ewing’s sarcoma, and cervical carcinoma (25, 26). Several clinical trials have also demonstrated its potential benefit in patients with NB (27, 28). These observations extend the known anti−tumor activities of HDAC inhibitors beyond direct suppression of cell proliferation and migration. In addition to intrinsic tumor cell cytotoxicity, emerging evidence highlights the critical immunomodulatory roles of HDAC inhibitors in reshaping the tumor immune microenvironment and reversing immune evasion. In the present study, we further demonstrate that SAHA attenuates HLA−E expression, a major checkpoint molecule that enables neuroblastoma cells to escape NK cell−mediated immune surveillance. By inhibiting the PERK/ATF4/CHOP pathway, SAHA not only reduces tumor cell growth and motility but also dampens HLA−E−dependent immune suppression. These findings support the concept that HDAC inhibitors such as SAHA act as dual−function agents, targeting both oncogenic signaling and immune escape programs, thereby strengthening their therapeutic potential in neuroblastoma.

To establish a model of HLA-E overexpression in NB, cell lines were cultured with IFN-γ *in vitro*. IFN-γ is a cytokine produced locally by tumor-infiltrating T cells and NK cells in the tumor microenvironment. In multiple cancer types, including ovarian cancer, acute myeloid leukemia, and melanoma, IFN-γ has been shown to induce HLA-E overexpression primarily via the STAT1 complex and GATA-1 signaling pathways (20, 21). In ovarian cancer, IFN-γ-induced HLA-E upregulation is associated with poor prognosis (29). In acute myeloid leukemia, IFN-γ promotes HLA-E expression and inhibits NK cell-mediated cytotoxicity through CD94/NKG2A signaling (30, 33). Similarly, in our study, IFN-γ upregulated HLA-E expression at both mRNA and protein levels in MYCN-amplified and non-amplified NB cell lines, establishing a robust model to simulate *in vivo* tumor cell immune evasion.

We further assessed HLA-E expression in NB cells treated with SAHA and found that SAHA reduced HLA-E expression at both the RNA and protein levels. SAHA significantly suppressed HLA-E mRNA expression regardless of IFN-γ stimulation. Furthermore, in cells with IFN-γ-induced HLA-E overexpression, the percentage of HLA-E(+) cells decreased from 71.8% to 44.8% after 24 h of SAHA treatment. These results suggest that SAHA is a potent inhibitor of HLA-E expression. However, HLA-E expression was not significantly reduced after 48 h of treatment, potentially due to the activation of feedback regulatory mechanisms or compensatory pathways that may interfere with SAHA’s effect—this warrants further investigation.

To examine the role of ERS in regulating HLA-E expression, we analyzed the data of patients with NB from the TARGET database and identified prognosis-related genes. HLA-E expression was significantly higher in the high-risk group compared to the intermediate-risk group. Through cross-referencing GO, KEGG, and GeneCards databases, six key genes were identified—*HLA-E*, *BCAP31*, *NGLY1*, *PERK* (*EIF2AK3*), *MAP3K5*, and *BAX*. Among these, *PERK* (*EIF2AK3*), *HLA-E*, and *BAX* were confirmed to be independent prognostic factors in NB. Prior research has shown that PERK, one of the three main ERS signaling pathways, activates ATF4 via phosphorylation, which in turn regulates the expression of the pro-apoptotic transcription factor CHOP (31, 34). These findings were validated using the GSE85047 dataset from the GEO database, further confirming the association between the PERK pathway and poor prognosis in patients with NB.

Previous studies have shown that SAHA can modulate ATF4 protein expression (32, 35). However, the link between SAHA, the PERK pathway, and ATF4-regulated targets like CHOP remains unclear. Our results demonstrated that SAHA downregulated the PERK pathway at the mRNA level, and both PERK and CHOP protein levels were reduced in MYCN-amplified NB cells. In NB cells with IFN-γ-induced HLA-E overexpression, SAHA significantly downregulated CHOP expression but not PERK or ATF4 protein levels. This may be due to negative feedback or compensatory mechanisms triggered by HLA-E overexpression, which could attenuate SAHA’s inhibitory effect on the PERK pathway. Further research is needed to clarify these mechanisms.

To further investigate the relationship between PERK-related pathways and HLA-E expression, Pearson correlation coefficients were calculated using the TARGET dataset. The analysis revealed a positive correlation between PERK, BAX, and HLA-E expression, suggesting a possible direct or indirect regulatory relationship between PERK pathway-associated genes and HLA-E expression.

In conclusion, this study demonstrated that SAHA downregulates HLA-E expression by inhibiting the PERK/ATF4/CHOP pathway. These findings provide new insight into the potential use of SAHA in controlling tumor proliferation, migration, and immune escape in NB.

## Data Availability

The data presented in the study are deposited in the Research Data Deposit (RDD) repository, accession number RDDB2025607117.

## References

[1] Coughlan D Gianferante M Lynch CF Stevens JL Harlan LC . Treatment and survival of childhood neuroblastoma: Evidence from a population-based study in the United States. Pediatr Hematol Oncol. (2017) 34:320–30. doi: 10.1080/08880018.2017.1373315, PMID: 29039999 PMC6764456

[2] Strother DR London WB Schmidt ML Brodeur GM Shimada H Thorner P . Outcome after surgery alone or with restricted use of chemotherapy for patients with low-risk neuroblastoma: results of Children’s Oncology Group study P9641. J Clin Oncol. (2012) 30:1842–8. doi: 10.1200/JCO.2011.37.9990, PMID: 22529259 PMC3383182

[3] Baker DL Schmidt ML Cohn SL Maris JM London WB Buxton A . Outcome after reduced chemotherapy for intermediate-risk neuroblastoma. N Engl J Med. (2010) 363:1313–23. doi: 10.1056/NEJMoa1001527, PMID: 20879880 PMC2993160

[4] Gooden M Lampen M Jordanova ES Leffers N Trimbos JB van der Burg SH . HLA-E expression by gynecological cancers restrains tumor-infiltrating CD8^+^ T lymphocytes. Proc Natl Acad Sci U.S.A. (2011) 108:10656–61. 10.1073/pnas.1100354108 PMC3127933 21670276

[5] Ferns DM Heeren AM Samuels S Bleeker MCG de Gruijl TD Kenter GG . Classical and non-classical HLA class I aberrations in primary cervical squamous- and adenocarcinomas and paired lymph node metastases. J immunotherapy Cancer. (2016) 4:78. doi: 10.1186/s40425-016-0184-3, PMID: 27895918 PMC5109766

[6] de Kruijf EM Sajet A van Nes JG Natanov R Putter H Smit VT . HLA-E and HLA-G expression in classical HLA class I-negative tumors is of prognostic value for clinical outcome of early breast cancer patients. J Immunol. (2010) 185:7452–9. doi: 10.4049/jimmunol.1002629, PMID: 21057081

[7] Talebian Yazdi M van Riet S van Schadewijk A Fiocco M van Hall T Taube C . The positive prognostic effect of stromal CD8+ tumor-infiltrating T cells is restrained by the expression of HLA-E in non-small cell lung carcinoma. Oncotarget. (2016) 7:3477–88. doi: 10.18632/oncotarget.6506, PMID: 26658106 PMC4823121

[8] Seliger B Jasinski-Bergner S Quandt D Stoehr C Bukur J Wach S . HLA-E expression and its clinical relevance in human renal cell carcinoma. Oncotarget. (2016) 7:67360–72. doi: 10.18632/oncotarget.11744, PMID: 27589686 PMC5341881

[9] Levy EM Bianchini M Von Euw EM Barrio MM Bravo AI Furman D . Human leukocyte antigen-E protein is overexpressed in primary human colorectal cancer. Int J Oncol. (2008) 32:633–41. doi: 10.3892/ijo.32.3.633, PMID: 18292941

[10] Zeestraten EC Reimers MS Saadatmand S Goossens-Beumer IJ Dekker JW Liefers GJ . Combined analysis of HLA class I, HLA-E and HLA-G predicts prognosis in colon cancer patients. Br J Cancer. (2014) 110:459–68. doi: 10.1038/bjc.2013.696, PMID: 24196788 PMC3899753

[11] Eugène J Jouand N Ducoin K Dansette D Oger R Deleine C . The inhibitory receptor CD94/NKG2A on CD8(+) tumor-infiltrating lymphocytes in colorectal cancer: a promising new druggable immune checkpoint in the context of HLAE/β2m overexpression. Mod Pathol. (2020) 33:468–82. doi: 10.1038/s41379-019-0322-9, PMID: 31409873

[12] Zhen ZJ Ling JY Cai Y Luo WB He YJ . Impact of HLA-E gene polymorphism on HLA-E expression in tumor cells and prognosis in patients with stage III colorectal cancer. Med Oncol. (2013) 30:482. doi: 10.1007/s12032-013-0482-2, PMID: 23377987

[13] Carlsten M Namazi A Reger R Levy E Berg M St Hilaire C . Bortezomib sensitizes multiple myeloma to NK cells via ER-stress-induced suppression of HLA-E and upregulation of DR5. Oncoimmunology. (2019) 8:e1534664. doi: 10.1080/2162402X.2018.1534664, PMID: 30713790 PMC6343814

[14] Pinto NR Applebaum MA Volchenboum SL Matthay KK London WB Ambros PF . Advances in risk classification and treatment strategies for neuroblastoma. J Clin Oncol. (2015) 33:3008–17. doi: 10.1200/JCO.2014.59.4648, PMID: 26304901 PMC4567703

[15] Qing G Li B Vu A Skuli N Walton ZE Liu X . ATF4 regulates MYC-mediated neuroblastoma cell death upon glutamine deprivation. Cancer Cell. (2012) 22:631–44. doi: 10.1016/j.ccr.2012.09.021, PMID: 23153536 PMC3510660

[16] Bingul M Arndt GM Marshall GM Black DS Cheung BB Kumar N . Synthesis and characterisation of novel tricyclic and tetracyclic furoindoles: biological evaluation as SAHA enhancer against neuroblastoma and breast cancer cells. Molecules. (2021) 26(19):5745. doi: 10.3390/molecules26195745, PMID: 34641289 PMC8510456

[17] Seneviratne JA Carter DR Mittra R . Inhibition of mitochondrial translocase SLC25A5 and histone deacetylation is an effective combination therapy in neuroblastoma. Int J Cancer. (2022) 152(7):1399–413. doi: 10.1002/ijc.34349, PMID: 36346110 PMC10953412

[18] van Esch EM Tummers B Baartmans V Osse EM Ter Haar N Trietsch MD . Alterations in classical and nonclassical HLA expression in recurrent and progressive HPV-induced usual vulvar intraepithelial neoplasia and implications for immunotherapy. Int J Cancer. (2014) 135:830–42. doi: 10.1002/ijc.28713, PMID: 24415578

[19] Borst L van der Burg SH Van Hall T . The NKG2A-HLA-E axis as a novel checkpoint in the tumor microenvironment. Clin Cancer Res. (2020) 26:5549–56. doi: 10.1158/1078-0432.CCR-19-2095, PMID: 32409305

[20] Gustafson KS Ginder GD . Interferon-gamma induction of the human leukocyte antigen-E gene is mediated through binding of a complex containing STAT1alpha to a distinct interferon-gamma-responsive element. J Biol Chem. (1996) 271:20035–46. doi: 10.1074/jbc.271.33.20035, PMID: 8702722

[21] Barrett DM Gustafson KS Wang J Wang SZ Ginder GD . A GATA factor mediates cell type-restricted induction of HLA-E gene transcription by gamma interferon. Mol Cell Biol. (2004) 24:6194–204. doi: 10.1128/MCB.24.14.6194-6204.2004, PMID: 15226423 PMC434230

[22] Mahaweni NM Bos GMJ Mitsiades CS Tilanus MGJ Wieten L . Daratumumab augments alloreactive natural killer cell cytotoxicity towards CD38+ multiple myeloma cell lines in a biochemical context mimicking tumour microenvironment conditions. Cancer Immunol Immunother. (2018) 67:861–72. doi: 10.1007/s00262-018-2140-1, PMID: 29500635 PMC5951903

[23] Kim I Xu W Reed JC . Cell death and endoplasmic reticulum stress: disease relevance and therapeutic opportunities. Nat Rev Drug Discov. (2008) 7:1013–30. doi: 10.1038/nrd2755, PMID: 19043451

[24] Kikuchi S Suzuki R Ohguchi H Yoshida Y Lu D Cottini F . Class IIa HDAC inhibition enhances ER stress-mediated cell death in multiple myeloma. Leukemia. (2015) 29:1918–27. doi: 10.1038/leu.2015.83, PMID: 25801913

[25] García-Domínguez DJ Hontecillas-Prieto L Rodríguez-Núñez P Pascual-Pasto G Vila-Ubach M García-Mejías R . The combination of epigenetic drugs SAHA and HCI-2509 synergistically inhibits EWS-FLI1 and tumor growth in Ewing sarcoma. Oncotarget. (2018) 9:31397–410. doi: 10.18632/oncotarget.25829, PMID: 30140378 PMC6101143

[26] Lee SY Huang Z Kang TH Soong RS Knoff J Axenfeld E . Histone deacetylase inhibitor AR-42 enhances E7-specific CD8^+^ T cell-mediated antitumor immunity induced by therapeutic HPV DNA vaccination. J Mol Med (Berl). (2013) 91:1221–31. doi: 10.1007/s00109-013-1054-9, PMID: 23715898 PMC3783646

[27] Phimmachanh M Han JZR O'Donnell YEI Latham SL Croucher DR . Histone deacetylases and histone deacetylase inhibitors in neuroblastoma. Front Cell Dev Biol. (2020) 8:578770. doi: 10.3389/fcell.2020.578770, PMID: 33117806 PMC7575710

[28] DuBois SG Granger MM Groshen S Tsao-Wei D Ji L Shamirian A . Randomized phase II trial of MIBG versus MIBG, vincristine, and irinotecan versus MIBG and vorinostat for patients with relapsed or refractory neuroblastoma: A report from NANT consortium. J Clin Oncol. (2021) 39:3506–14. doi: 10.1200/JCO.21.00703, PMID: 34270348 PMC8547934

[29] Zheng H Guan X Meng X Tong Y Wang Y Xie S . IFN-γ in ovarian tumor microenvironment upregulates HLA-E expression and predicts a poor prognosis. J Ovarian Res. (2023) 16:229. doi: 10.1186/s13048-023-01286-z, PMID: 38007483 PMC10675946

[30] Dutta S Ganguly A Chatterjee K Spada S Mukherjee S . Targets of immune escape mechanisms in cancer: basis for development and evolution of cancer immune checkpoint inhibitors. Biology. (2023) 12(2):218. doi: 10.3390/biology12020218, PMID: 36829496 PMC9952779

[31] Rozpedek W Pytel D Mucha B Leszczynska H Diehl JA Majsterek I . The role of the PERK/eIF2α/ATF4/CHOP signaling pathway in tumor progression during endoplasmic reticulum stress. Curr Mol Med. (2016) 16:533–44. doi: 10.2174/1566524016666160523143937, PMID: 27211800 PMC5008685

[32] Wolf IM Fan Z Rauh M Seufert S Hore N Buchfelder M . Histone deacetylases inhibition by SAHA/Vorinostat normalizes the glioma microenvironment via xCT equilibration. Sci Rep. (2014) 4:6226. doi: 10.1038/srep06226, PMID: 25228443 PMC4165982

[33] Lee N Llano M Carretero M Ishitani A Navarro F López-Botet M . HLA-E is a major ligand for the natural killer inhibitory receptor CD94/NKG2A. Proc Natl Acad Sci U.S.A. (1998) 95:5199–204. doi: 10.1073/pnas.95.9.5199, PMID: 9560253 PMC20238

[34] Zhen Z Yang K Ye L You Z Chen R Liu Y . HLA-E inhibitor enhances the killing of neuroblastoma stem cells by co-cultured dendritic cells and cytokine-induced killer cells loaded with membrane-based microparticles. Am J Cancer Res. (2017) 7:334–45. PMC5336506 28337381

[35] Tang XX Shimada H Ikegaki N . Clinical relevance of CD4 cytotoxic T cells in high-risk neuroblastoma. Front Immunol. (2021) 12:650427. doi: 10.3389/fimmu.2021.650427, PMID: 33968044 PMC8101497

